# Factors Related to Smoking and Perceptions of a Behavioral Counseling and Messenger Service–Delivered Smoking Cessation Intervention for People With HIV in China: Qualitative Study

**DOI:** 10.2196/35923

**Published:** 2022-10-12

**Authors:** Shanyin Yang, Jiegang Huang, Li Ye, Jianyan Lin, Zhiman Xie, Baodong Guo, Yanjun Li, Bingyu Liang, Zhigang Zheng, Karsten Lunze, Abu S Abdullah, Hao Liang, Lisa M Quintiliani

**Affiliations:** 1 Section of General Internal Medicine Boston Medical Center Boston, MA United States; 2 Guangxi Key Laboratory of AIDS Prevention and Treatment School of Public Health Guangxi Medical University Nanning, Guangxi China; 3 Collaborative Innovation Centre of Regenerative Medicine and Medical BioResource Development and Application Co-constructed by the Province and Ministry Nanning, Guangxi China; 4 The Fourth People's Hospital of Nanning Nanning, Guangxi China; 5 Department of Medicine Boston Univeristy School of Medicine Boston, MA United States; 6 Life Science Institute Guangxi Medical University Nanning, Guangxi China

**Keywords:** mobile health, mHealth, China, smoking, smoking cessation, HIV, qualitative research, SMS text messages, WeChat

## Abstract

**Background:**

China, where half of the adult male population smoke tobacco, has one of the highest global burdens of smoking. Smoking rates are even higher among people with HIV. People with HIV can be affected by smoking in multiple ways, including more severe HIV-related symptoms and worse antiretroviral therapy treatment outcomes. However, smoking cessation services targeted for people with HIV are not routinely integrated into HIV care in China. Given the widespread mobile phone ownership, an exploration of factors related to smoking among people with HIV in China who smoke could inform the design and implementation of mobile smoking cessation interventions that target the needs of this vulnerable population.

**Objective:**

This study aims to explore the perspectives of smoking, barriers and facilitators to quitting, and perceptions related to a smoking cessation intervention delivered through behavioral counseling sessions and brief daily messenger service (WeChat)–delivered messages.

**Methods:**

We recruited people with HIV from the People’s 4th Hospital of Nanning, Guangxi, China, and conducted semistructured face-to-face interviews. All interviews were audio-recorded, transcribed verbatim in Chinese, and translated into English for data analysis. We conducted a thematic analysis using a codebook, which was guided by a team-based consensus approach to identify 5 main themes. We also explored themes according to the demographic groups.

**Results:**

A total of 24 participants were enrolled in the study. The mean age was 37.2 (SD=13.5) years. The participants had lived with HIV for a mean of 2.4 years. The majority were male (18/24, 75%) and lived in urban or metropolitan settings (19/24, 79%). We identified five main themes: variable knowledge of the harms of smoking, both related and unrelated to HIV; willpower perceived as the primary quitting strategy; a duality of the effect of social factors on quitting; perceptions about optimal features of the smoking cessation intervention (eg, messages should be brief and most frequent during the first few weeks); and the largely negative impact of their HIV diagnosis on smoking behaviors. In addition, some themes differed according to participant demographic characteristics such as age, sex, and education level.

**Conclusions:**

We identified barriers to and facilitators of smoking cessation among people with HIV in China by conducting semistructured qualitative interviews. Owing to the adverse impact of smoking on HIV outcomes, targeting cessation interventions to the unique needs and preferences of people with HIV in China may be needed to increase the effectiveness of future interventions. A pilot clinical trial will be conducted in the future to evaluate this behavioral counseling and brief daily messenger service (WeChat)–delivered messages approach among people with HIV who smoke in China.

## Introduction

### Background

Tobacco use is one of the most important preventable causes of premature death worldwide. In 2018, approximately 50.5% of adult males in China and 2.1% of adult females in China smoked cigarettes [1]. Smoking is more prevalent among people with HIV compared with the general population in China. For instance, a sample of males with HIV from Yunnan province in 2012 had a smoking prevalence of 92% [2]. In addition, the same sample showed that a majority (67.6%) of people with HIV who smoked in China were heavy smokers, smoking 20 cigarettes per day [2]. People with HIV are affected by tobacco in multiple ways: more severe HIV-related symptoms [2-4]; worse treatment outcomes, in part owing to lower antiretroviral therapy adherence [4]; and higher rates of tobacco-related comorbidities such as lung cancer, cervical cancer, and pulmonary disease [4,5]. In China, people with HIV who smoke experience high degrees of stigma, prejudice, and discrimination [6,7]. People with HIV who experience HIV stigma can have high psychosocial distress and low self-esteem and resilience [8]. To deal with HIV-related stigmatization and depression [9], both of which are highly prevalent among people with HIV and have adverse effects on HIV care engagement [10], they may choose to engage in smoking behavior as a coping strategy [8]. Owing to the harmful nature of smoking with negative implications on the course of HIV infection and success of treatment, targeted smoking cessation interventions are needed.

Although there is a large body of research on smoking cessation interventions for the general population [11,12], smoking cessation interventions specifically targeting people with HIV in China are lacking and have not been routinely integrated into clinical HIV services. This specific population has high motivation to engage in smoking cessation interventions to improve health outcomes [13,14]. A few studies have been published about smoking cessation efforts among people with HIV in China who smoke [13,14]; however, these studies are limited to cross-sectional surveys examining motivations for cessation, instead of evaluating cessation interventions [13,14]. An in-depth understanding of the factors related to smoking behaviors and preferences would present opportunities to design culturally targeted smoking cessation interventions for people with HIV who smoke in China.

Mobile phones are a widely used mode of delivery for behavioral interventions because they are readily accessible in many populations and have the potential for widespread use. In China, interventions using mobile technologies for smoking cessation such as SMS text messaging and mobile apps have demonstrated efficacy in smoking cessation [15,16]. In particular, in China, the WeChat messenger service platform has demonstrated efficacy in changing behaviors across a range of behaviors and health conditions [17-21], including among people with HIV for decreasing depressive symptoms and improving medication adherence [19,20].

### Objectives

The goal of this study was to identify factors related to smoking and cessation among people with HIV in China who were current or former smokers and to explore their perspectives on how to modify a draft of *Quit for Life*, an 8-week intervention that combined 4 counseling sessions and brief daily messenger service (WeChat)–delivered messages. *Quit for Life* was based on a previous intervention, which consisted of cessation and nicotine replacement therapy (NRT) adherence counseling and did not include WeChat messages, tested among Chinese patients living in Hong Kong with erectile dysfunction in a randomized controlled trial conducted from 2004 to 2007. The previous intervention was shown to be effective; the intervention group participants were more likely to quit smoking compared with the control group [22]. On the basis of these efficacy findings, we selected this intervention and decided to boost its potential effectiveness and reach with mobile technology (ie, WeChat messaging). We focused on exploring participants’ perspectives on the new component of the intervention (ie, WeChat messaging) and how to modify all intervention content for people with HIV.

## Methods

### Study Setting and Recruitment

The study was conducted at the People’s 4th Hospital of Nanning, Guangxi, China. The Guangxi Zhuang Autonomous Region (Guangxi) is located in the southwest region of China. From 2013 to 2015, HIV prevalence in Guangxi increased from 6.6% to 11.2% [23]. In 2018, Guangxi reported more than 50,000 HIV cases in 1 year, ranking it third for the highest number of HIV cases among all provinces in China [24]. From 2003 onward, the Chinese government started providing HIV prevention services and free antiretroviral therapy to encourage individuals to participate in screening and treatment [24]. People’s 4th Hospital of Nanning is the only grade A (the highest classification) tertiary hospital (comprehensive referral hospitals) in Nanning city.

To recruit participants, research assistants contacted individuals who received HIV treatment at the outpatient HIV clinic or who were hospitalized in 2021. The eligibility criteria were as follows: aged ≥21 years, HIV seropositive, current or former smokers, receiving antiretroviral therapy, receiving HIV care at the clinic at the 4th People’s Hospital of Nanning, and speaking Mandarin Chinese. Exclusion criteria were as follows: any serious health problems that preclude participation, only smoke tobacco products other than cigarettes (ie, cigars and electronic devices), or inability to give consent.

### Data Collection

A graduate student interviewer, trained by an investigator on the Guangxi Medical University team experienced with qualitative methods, conducted all interviews face-to-face in Mandarin Chinese. A health care provider from the HIV clinic (ie, physicians and nurse practitioners) was present during the interview and answered the participants’ medical questions, if any. All interviews were audio-recorded. Before the interview, the interviewer administered a survey assessing demographic information such as age, sex, ethnicity, education level, current smoking behaviors, and HIV status (self-reported diagnosis, diagnosis and time since diagnosis were later verified in the health record). The interviewer then conducted the interview following a semistructured guide with open-ended questions (Multimedia Appendix 1) consisting of the following topics: smoking patterns and motivations; knowledge of general health impacts of smoking, second-hand and third-hand smoke, and e-cigarettes; knowledge of the impact of smoking on HIV and disease prognosis; and experiences with previous attempts to quit and perceived facilitators and barriers to quitting. The interviewer briefly described the draft *Quit for Life* intervention, including reading 4 sample WeChat messages, and then explored preferences for delivery modality, duration of contact, frequency of contact, and desired content. Finally, participants had the opportunity to provide additional comments. Participants received compensation (equivalent to US $15) for completing the onetime interview.

### Data Analysis

The research team entered participants’ demographic information using the Research Electronic Data Capture tools (Vanderbilt University) hosted at Boston University [25]. We computed the frequencies of demographic variables to characterize the study sample. Investigators from China transcribed the interview recordings verbatim in Mandarin, and a bilingual second research assistant who is a native Mandarin speaker then translated the transcriptions into English. The research team in China reviewed the transcripts for validation purposes. All data analyses were performed using the final set of English language transcripts. Using a thematic coding process [26], the bilingual research assistant systematically reviewed all transcripts, generated and noted initial ideas, and developed a preliminary codebook. In consensus with another investigator with expertise in qualitative methods, we modified and refined the codes and subcodes. All transcripts were then coded using the final codebook, with NVivo version 12 (QSR International) as the data management platform. Two team members grouped the codes into themes. We then held 2 joint meetings with research team members from the Guangxi Medical University team in China (including the original interviewers) to collect their feedback about the themes and cultural context. During the joint meetings, we decided to explore demographic (age, sex, and education level) differences in our coding. As we did not address demographic differences directly in our interview guide, we categorized them as exploratory themes. At the end of this process, we developed a final version of the thematic categories.

### Ethical Considerations

The ethics approval for this study was obtained from the institutional review boards of the Boston Medical Center (Institutional Review Board number: H-40111) and the Guangxi Medical University.

## Results

### Overview

A total of 24 participants were enrolled; the majority of participants were male (18/24, 75%) and from outpatient HIV units (19/24, 79%; Table 1). Interviews were conducted either individually (18 interviews) or in pairs (3 interviews). The average duration of the interviews was approximately 30 minutes. We identified 5 thematic categories (Table 2) and explored themes among demographic categories (Multimedia Appendix 2).

**Table 1:** Sociodemographic characteristics of smokers and former smokers with HIV in China participating in qualitative interviews (N=24).

Characteristics		Value
Age (years), mean (SD)		37.3 (13.5)
**Sex, n (%)**		
	Male	18 (75)
	Female	6 (25)
**Education level^a^, n (%)**		
	Primary or lower	2 (8)
	Lower secondary	8 (33)
	Upper secondary	8 (33)
	Tertiary or higher	4 (17)
	Missing	2 (8)
**Ethnicity,** **n (%)**		
	Han	13 (54)
	Zhuang	10 (42)
	Other	1 (4)
**Marital status,** **n (%)**		
	Divorced	4 (17)
	Married	9 (37)
	Unmarried	11 (46)
**Smoking status,** **n (%)**		
	Current	18 (75)
	Former	6 (25)
Smoking duration (years), mean (SD)		18.6 (13.6)
Smoking amount (cigarettes per day), mean (SD)		20.9 (15.4)
Duration of HIV diagnosis (years), mean (SD)		2.4 (2.7)
Duration of ART^b^ (years), mean (SD)		2.1 (2.8)

^a^Primary or lower education level in China is equivalent to grades 6 or lower in the United States; lower secondary education level in China is equivalent to grades 7 to 9 in the United States; upper secondary education level in China is equivalent to high school grades 10 to 12 or vocational training in the United States; and tertiary education level in China includes junior college, undergraduate, and graduate school.

^b^ART: antiretroviral therapy.

**Table 2:** Frequently noted themes with supporting codes and example quotations of smokers and former smokers with HIV in China.

Theme and parent code		Subcode	Additional subcode		Quotations
**Theme 1: Knowledge on the harms of smoking and personal risk perception**					
	Knowledge	Source of knowledge		—^a^	“I watched TV ads saying that smoking is bad and harmful to your health.” [M^b^, 37, C^c^] “[I learned smoking would impact my health from] Science, doctors, and people in the society.” [M, 33, C]
	Knowledge	Health effects		—	“Every year when I do physical examination, my lungs appear to be influenced by smoking to some extent...” [M, 24, Q^d^] “...I know it’s bad for health. But I don’t know the details on how and what exactly does it impact.” [F^e^, 38, C]
	Knowledge	Effects on HIV		—	“I don’t think there’s a relationship between smoking and HIV infection. It may affect treatment.” [M, 48, Q] “There’s certainly a damage to the body, but I don't know much about it.” [M, 25, C]
	Knowledge	Second- and third-hand smoke		—	“I know second-hand smoke, but not third-hand smoke.” [M, 46, C]
	Barriers and motivators to quitting	Barriers to quit		Risk perception	“...people say it’s carcinogenic and affects my lungs. However, since I’m smoking, I shouldn’t think of those things too much.” [M, 21, C]
	Barriers and motivators to quitting	Daily stress		—	“Yes, it is possible [to not relapse if there were no recent stress from work].” [M, 24, C]
	Smoking behavior	Feelings associated with smoking		—	“When I talk about happy things, I smoke more cigarettes than usual.” [M, 25, C] “I smoked more when I was moody or unhappy and smoked less when I had lighter moods. Having stress also made me smoke more.” [M, 71, Q]
**Theme 2: Willpower as a primary quitting strategy**					
	Strategies	Willpower		—	“...NRT^f^ or other medications are only facilitators. One’s own willpower is the most important and the most crucial.” [M, 46, C]
	Strategies	Gradually decrease		—	“I used to smoke, but it decreased slowly after I got sick, and then I really stopped smoking. When others give me cigarettes, I refused by saying I don't smoke anymore. I tend to avoid occasion involving drinking and socializing as much as possible.” [M, 43, Q]
	Strategies	Suddenly decrease		—	“I think it’s useless to slowly reduce the amount. If you want to resist the craving, give it up all at once, and you’ll have better chance of quitting successfully.” [M, 48, Q]
	Knowledge	Available cessation resources		—	“If I’m sick and the doctor says I shouldn’t smoke or drink, then I won’t smoke.” [M, 21, C]
	QFL^g^ intervention	Useful		Initial interest in joining	“If I see it [smoking cessation counseling offered at outpatient clinic], I will go check it out and learn more about it.” [M, 29, C]
	Quit attempts	Perceptions of cessation resources		Positive	“It can be difficult to refrain from smoking without medications or other help.” [M, 37, C]
	Quit attempts	Perceptions of cessation resources		Negative	“No, there is no need [for other cessation resources]. I can rely on myself to quit.” [M, 30, C]
	Additional concepts	E-cigarettes		Not useful	“I smoked e-cigarettes and it was completely useless. So I shifted back to smoking cigarettes. After I tried smoking cessation, I smoked again and with greater quantity” [M, 48, Q]
	Additional concepts	E-cigarettes		Useful	“...I’ve used it [e-cigarettes] before. It can replace real cigarettes for a period of time.” [M, 24, C]
**Theme 3: Duality of social factors**					
	Smoking behavior	Reasons to start smoking		Curiosity	“I was bored...And I was curious about the feeling of smoking, so I bought cigarettes to try.” [M, 21, C]
	Smoking behavior	Reasons to start smoking		Social	“I went out with friends, and they handed me the cigarettes.” [F, 22, C]
	Barriers and motivators to quitting	Barriers to quit		Social pressure	“After I was diagnosed with HIV, I stopped smoking for half a year and then started again. It was during banquets...I was given cigarettes...and then became addicted to it again after a while.” [M, 25, C]
	Smoking behavior	Patterns of smoking		With whom	“...when I’m hanging out with my friends, or when my friends give me cigarettes.” [F, 44, C]
	Smoking behavior	Patterns of smoking		Setting	“There is no such feeling [influencing others’ health] because most people smoke.” [M, 50, C] “When there are other people together I won’t smoke in cars. I choose to park on the side of the road, smoke and then leave. If I’m alone driving, I smoke with my windows open.” [M, 37, C]
	Quit attempts	Details about attempts		—	“...I was thinking too much, I couldn’t accept [the HIV diagnosis], so I started smoking again.” [M, 30, C]
	Barriers and motivators to quitting	Motivators to quit		Social support	“Quitting together is definitely better than quitting alone, no matter if it’s having one person as the leader or keeping track of each other’s progress.” [M, 46, C] “The encouragement of my family [helped with smoking cessation].” [M, 43, Q]
	Barriers and motivators to quitting	Motivators to quit		Family pressure	“My family didn’t allow me to smoke, doctors didn’t allow me to smoke either.” [M, 58, C] “When first I stopped smoking, I would go to places where I used to put cigarettes, but there was no way I could find them.” [M, 58, C]
	Barriers and motivators to quitting	Motivators to quit		Positive impacts on others	“[An advice for other smokers:] For the health of people around you, try your best to reduce the frequency of smoking, and be mindful of your surrounding.” [M, 33, C] “Because you have the responsibility to take care of the family, things you do are closely linked to the future of the family.” [M, 24, Q]
	Barriers and motivators to quitting	Motivators to quit		Social influence and stigma	“Smoking is not accepted by the society, it has no social status. Especially you are in a car with a woman. It is not good and you should not smoke continuously at least in terms of human nature...” [M, 46, C]
	Knowledge	Second- and third-hand smoke		—	“My family used to blame me for inhaling my second-hand smoke, but I can't control it.” [M, 48, Q]
**Theme 4: Impact of HIV diagnosis**					
	HIV diagnosis	After HIV diagnosis		—	“It probably does. I tried to control myself. After I was diagnosed with HIV, I did not touch a cigarette for at least two years. However, before Lunar New Year last year, I gradually started again. I smoked a little, then a little more, and then got used to it.” [F, 36, C] “Before, I smoked one pack in two days, after I learned it [HIV diagnosis], I decreased the amount.” [M, 29, C] “I don’t work anymore...Mainly because some jobs need a health certificate. Since I have HIV infection, I cannot apply for the health certificate.” [M, 25, C]
	Additional concepts	ART^h^ use		—	“After I was diagnosed with HIV, I stopped the quitting thoughts. I think smoking only has minor influence toward human body. I am taking medications everyday now, and that cause more problems.” [M, 46, C]
	Barriers and motivators to quitting	Motivators to quit		Health benefits and physical changes after quiting	“If I’m pregnant, I will definitely quit. Reminding someone like me that they have something important to concern [pregnancy etc.].” [F, 44, C] “My HIV infection and severe lung infection caused me to be hospitalized. I quitted smoking when I was being hospitalized. After I returned home, I didn’t smoke anymore...family members didn’t allow me to smoke either. I almost died.” [M, 48, Q]
	Barriers and motivators to quitting	Barriers to quit		Daily stress	“...I didn’t [smoke when hospitalized]. I started smoking three months after I was discharged...” [ F, 25, C] “...I was thinking too much, I couldn’t accept [the HIV diagnosis], so I started smoking again.” [M, 30, C]
	QFL intervention	Length and time of intervention		—	“Many people...are having a hard time accepting the fact that they’re diagnosed with HIV infection. If you talk to them about smoking cessation, they won’t be interested and won’t be willing to spend too much time on it.” [M, 24, Q]
**Theme 5: QFL intervention modification**					
	QFL intervention	Modality		—	“I will read WeChat messages. I usually read WeChat but not text messages.” [M, 24, C] “[WeChat is effective for the intervention, but] don’t construct a WeChat group [vs. private messaging]. That involves personal privacy and no one wants others to know about their condition.”[ M, 46, C] “A little bit. I am not very familiar with WeChat.” [M, 71, Q] “I might not answer all the calls...If the number is not from Nanning City, I won’t pick up.” [M, 58, C]
	QFL intervention	Content		—	“...I think it’s good to not send any messages at all...you are reminding me that ‘I am a smoker’.” [F, 36, C] “...most mobile messages...are advertisements so I get annoyed and don’t even read them.” [M, 48, Q]
	QFL intervention	Length and time of intervention		—	“When we have to wait in line to see a doctor, 40 minutes is a bit long, 20 minutes would be appropriate.” [M, 43, Q]
	QFL intervention	Setting		—	“It’s fine if I talk to someone face-to-face when I’m picking up my medications.” [M, 29, C]
	QFL intervention	Frequency		—	“The most important period for quitting in the first two weeks...If you can resist your cravings for the first two weeks, then you can stop smoking.” [M, 48, Q]

^a^Dashes represent when there are no additional subcodes.

^b^M represents participants who report to be male.

^c^C represents participants who report to be current smokers.

^d^Q represents participants who report to be former smokers and have quit smoking.

^e^F represents participants who report to be female.

^f^NRT: nicotine replacement therapy.

^g^QFL: *Quit for Life*.

^h^ART: antiretroviral therapy.

### Theme 1. Some Awareness of Smoking Harms in General, Yet Low Personal Risk Perceptions of Smoking and Impacts on HIV Treatment Care Outcomes

Most participants understood that smoking harms their lungs and health, but to different extents. For example, some participants knew that smoking leads to a worsening of their HIV treatment outcomes. Other participants had a limited understanding of the harms from smoking on their own health; for example, a person noted the following: “at least at the moment smoking does not cause any harm for me so far.” A participant expressed that as they had not heard anything from authoritative sources, they would still “take a chance” and think smoking is not harmful. When asked about the harm of smoking and the harm of second- and third-hand smoke, most participants had a general awareness from their medical providers, from television, or through social media, but they lacked specific knowledge of these terms.

Participants saw a close connection among smoking and mood, emotions, and mental well-being. About half of the participants agreed that emotions were closely linked with cravings to smoke. Several smokers mentioned that they “smoked more when [they were] moody or unhappy and smoked less when [they] had lighter moods.” Workplaces were often mentioned as environments that induced stress and anxiety, leading to smoking to calm down and escape.

### Theme 2: Willpower Was Identified as a Primary Quitting Strategy, With Less Awareness and Trust in Other Resources

When asked about known strategies for quitting, participants most often mentioned individual effort, determination, and personal willpower; for example: “quitting smoking mainly depends on [one’s] self-control of the impulse to smoke.” A few participants had tried or thought about decreasing the number of cigarettes smoked gradually, whereas others tended to decrease suddenly. Participants had limited awareness of available smoking cessation resources other than they “can rely on [themselves] to quit.” However, participants were willing to trust their medical providers. Specifically, a participant pointed out the following: “If I see it [smoking cessation counseling offered at outpatient clinic], I will go check it out and learn more about it.” Among those who understood the potential of e-cigarettes as a smoking cessation aid, almost all considered them “completely useless” and felt that e-cigarettes would worsen their cravings for cigarettes. For the subset of participants who acknowledged the availability of cessation resources, most thought that these methods would not be useful. A few participants also mentioned that candies and snacks were the distraction methods they used to quit smoking.

### Theme 3: The Duality of Social Factors: Both Discouraging and Encouraging for Quitting

Most participants reported that they started smoking because of curiosity and social pressure. A participant noted that she started smoking when “[she] went out with friends, and they handed [her] the cigarettes.” Social occasions where they “were given cigarettes” prevented the participants from quitting. Several participants mentioned that they often smoked with friends; relapse after a quit attempt tended to occur because they saw friends smoking. Smoking with friends was considered a necessary part of social interaction. Regarding societal perceptions, some participants expressed a few negative connotations about smoking in public places because most people do the same. Smoking in cars was also common when passengers were smokers, but participants refrained from smoking with nonsmoking passengers. Participants noted that social stigma about their HIV diagnosis induced stress, and smoking was perceived as needed to alleviate this stigma-induced stress.

By contrast, participants also mentioned social pressure and peer support as facilitators to quitting. This included support from family and friends, supervision, and the benefits of quitting together. A participant mentioned the following: “the encouragement of my family [helped with smoking cessation].” Other social facilitators to quitting included awareness that their smoking behavior harmed their loved ones (such as children) or had a negative influence on their family. In some instances, participants felt that family members blamed them for creating harm through second- and third-hand smoke, and family members would pressure them to quit. Social stigma is believed to play a role in facilitating quitting. There was a sense that “smoking is not accepted by the society,” especially when there are others (such as women) nearby.

### Theme 4: HIV Diagnosis Raised Awareness of Participants’ Own Health but Also Induced Stress and Anxiety

When asked about changes after receiving the HIV diagnosis, some participants decided to, or felt the necessity to, quit or reduce the number of cigarettes smoked immediately after the diagnosis or because of other health conditions. Participants expressed fear and sadness when they first learned about their HIV diagnoses. Some participants described the effect of hospitalization owing to severe lung infection as pivotal to quitting (“family members [prohibited him] from smoking”), whereas others described being forced to quit smoking while being hospitalized. Those people mostly “started smoking again after [they were] discharged from the hospital” owing to stress from life or work. Similarly, several participants “couldn’t accept [the HIV diagnosis], so [they] started smoking again.” Having an HIV diagnosis induced societal pressure and stigma toward patients, which was felt to prevent engagement in daily tasks. For instance, a few participants reported having trouble finding a job because some jobs required a health certificate that cannot be obtained with a HIV diagnosis. Therefore, many participants returned to smoking because of reasons such as dependency on nicotine, stress, and social pressure.

The participants were hesitant to use NRT, such as patches or gum, because they were not willing to take additional medications. Ultimately, for some participants, concerns about the side effects of antiretroviral treatment medications overshadowed the potential benefit of using NRT (eg, a participant thought the pain caused by antiretroviral treatment medications would cause more problems than smoking).

### Theme 5: Quit for Life Intervention Modification

Participants were asked about their perceptions of the existing draft of the *Quit for Life* intervention. Almost all participants preferred using WeChat as the primary messenger service platform to receive messages, as it is less likely to be ignored and considered as scam messages (compared with SMS text messaging, which are often scams). Although participants thought WeChat was an acceptable form of communication, one participant thought that messages would act as a constant reminder of the idea that “I am a smoker” and could potentially backfire. A participant who was less familiar with WeChat as a messenger service platform thought that they would still read all the WeChat messages they received. Participants were concerned with using WeChat group (vs direct one-on-one WeChat messaging) because “[WeChat groups] involve personal privacy and no one wants others to know about their condition.” Some participants suggested sending more messages during the first 2 weeks of the intervention, as that period is when cravings occur more often and with higher intensity. For counseling sessions, participants thought that calls originating from a reliable area code in Nanning city would have a higher chance of being picked up. Many participants agreed that scheduling counseling sessions to coincide with picking up HIV medications was desirable. Participants endorsed face-to-face counseling at the HIV clinic and suggested that the counseling sessions be brief (10-20 minutes).

### Exploratory Theme: Demographic Differences in Perceptions of Smoking and the Decision to Quit Smoking

Table 2 presents exploratory themes that were summarized from the interview results. When comparing younger (<30 years) and older (>30 years) participants, responses differed in that older participants tended to quit because of health concerns or social stigma; in contrast, younger participants expressed unwillingness to quit because of low-risk perception despite knowing the harms of smoking. Compared with older participants, younger participants were more likely to smoke when hanging out with friends. We also explored comparisons between male and female smokers. Some female participants considered smoking and NRT to be more harmful to women compared with men, and pregnancy would be an event that would motivate them to quit (a female participant noted HIV by itself would not stop her from smoking). Male participants mentioned that societal perceptions of masculine ideals, familial responsibilities, and being dissuaded from expressing negative emotions act as barriers to quitting. Regarding educational background, risk perception tended to be low among participants with both lower and higher levels of education. However, those with higher levels of education had heard of second-hand and third-hand smoke and could explain these terms more often, whereas those with lower educational levels had more limited knowledge of these terms.

## Discussion

### Principal Findings

This qualitative study is one of the first to explore the salient perspectives of smoking and quitting topics including smoking patterns, facilitators and barriers to cessation, perceptions of cessation resources, and HIV stigma in people with HIV who smoke in China. In general, participants were willing to quit if their health was affected by smoking or were told to quit by their health care providers. This finding is consistent with quantitative studies that found that people with HIV who smoke in China have a higher likelihood of reporting willingness to quit than the general population of smokers without HIV [13,14].

### Comparison With Prior Work

First, similar to previous studies examining smoking cessation strategies among smokers in China, participants identified willpower as a primary quitting strategy in this study [27-29]. A lack of awareness of available NRT combined with a lack of smoking cessation interventions designed specifically for people with HIV who smoke in China are barriers for this population to seek external help [13,28]. At the same time, limited knowledge prevented some participants from understanding and acknowledging the usefulness of the smoking cessation services. Future interventions could promote the availability and usefulness of NRT for people with HIV who smoke.

Second, perceived stress was also a factor across several themes (eg, themes 1, 3, and 4). This finding is similar to previous studies conducted among teenagers and adults in China and Hong Kong, where peer pressure induced stress and need to conform, so that one could feel like they belonged to a social group—factors that could be addressed when designing cessation intervention content [27,30,31]. This barrier is more pronounced when considering China’s collectivistic culture, which focuses on social acceptance and the perception of cigarettes as a necessary social connection tool [32]. Stress was also perceived to be closely linked to the workplace. People in China traditionally view work as an integral part of their life as they work to support their family, even more so than their Western counterparts [33]. Stressful events during work include intense competition between coworkers within the same vocation [34], along with long working hours for many occupations [35]. Higher levels of work stress have been shown to result in a lower quality of life and higher levels of somatic symptoms and burnout [30,35,36]. In addition, people with HIV endure stress from the risk of involuntary serostatus disclosure, health status confidentiality, risk of viral transmission to others, fear of stigma and discrimination, and impairment of physical functioning [37]. For people with HIV in China, the compounded effect of stress from workplace environments and learning of their HIV diagnosis may lead to symptoms of depression and anxiety and prompt risk behaviors such as tobacco use, substance use, and unhealthy alcohol use [8,37-40], with a high likelihood of engaging in multiple risk behaviors [8,38,40]. Thus, future work should address the mental health needs of people with HIV who smoke in China, help this population develop strategies to cope with stressors, and consider multiple risk factor interventions.

Third, regarding the exploratory demographic differences with respect to sex, we found several female participants who perceived NRT as more dangerous for them compared with men. This aligns with previous research among women who were pregnant or planning to become pregnant [41]. Especially for women during pregnancy, taking NRT was perceived to be a burden because of other medications they had to take [41]. Future qualitative studies should consider directly investigating this exploratory finding among a larger sample of women in addition to other demographic differences (age and education level).

In addition, the draft telephone- and messenger-delivered smoking cessation intervention, *Quit for Life*, will be modified based on these qualitative results. For example, doctors and nurses were found to be common, trusted, and motivating sources of information on the importance of cessation and available resources. Therefore, for the *Quit for Life* intervention, interactions between medical providers and participants can enhance this trust-based relationship and may improve effectiveness and adherence to the study protocol [19]. One way to build trust is to have participants attend face-to-face initial counseling sessions in the HIV clinic with a counselor. As another example, concerns about the health of others, social disapproval, and stress were mentioned as motivators to quit, which is aligned with motivators from prior research, where those who quit endorsed health concerns, family disapproval, and being an example for their children [32]. Therefore, WeChat messages and counseling sessions will incorporate content related to health impacts, social influences of smoking, and formulating treatment plans to help the participant resolve barriers. Intervention content will also focus on the harms of smoking among people with HIV, specifically to respond to variable knowledge on this topic. Counseling sessions will follow motivational interviewing techniques.

Similar to previous research on telephone-delivered interventions in China, participants preferred the WeChat messenger service platform rather than other mobile service platforms [12,19,21,28,42]. Messages from the *Quit for Life* intervention will use short informative phrasing, be sent through a designated study account, and have a higher frequency at the start of the intervention period when cravings are thought to be the highest. Similar to our findings, previous cessation research in China has also reported generally low interest in cessation services and resources [27]. Given its efficacy for cessation [5], the *Quit for Life* intervention will provide widely available NRT gum at no cost for a time-limited duration. A pilot clinical trial will be conducted to test the preliminary efficacy of the *Quit for Life* intervention (WeChat messaging plus behavioral counseling) in addition to NRT plus educational booklets versus NRT plus educational booklets only on smoking cessation at 12 weeks.

### Limitations

Although we obtained in-depth information from our sample, there was an imbalance across demographic categories. For example, the number of female participants was low (reflecting the lower proportion of Chinese females who smoked in general). We did not conduct additional methods such as member checking; however, our multiteam collaboration provided regular input into the analysis, which lends credibility. In addition, more recent surveillance data on national smoking trends among people with HIV in China are needed to promote targeted cessation efforts.

### Conclusions

We examined the perspectives of people with HIV in China about smoking patterns, attitudes toward quitting, and HIV stigma to inform the development of a smoking cessation intervention to be delivered through counseling sessions and brief messenger service–delivered messages, specifically targeting this vulnerable population. In the future, the modified *Quit for Life* intervention will be evaluated in a pilot clinical trial among people with HIV who smoke in China recruited from the People’s 4th Hospital of Nanning in Nanning, Guangxi, China. If effective, the *Quit for Life* intervention has the potential to be implemented on a wider scale for the people with HIV who smoke in China, in part using mobile messenger service platforms.

## Appendix

Multimedia Appendix 1 Qualitative interview guide with open-ended questions.

Multimedia Appendix 2 Frequently noted themes with supporting codes and example quotations of smokers and former smokers with HIV in China.

## Abbreviations

NRT: nicotine replacement therapy
