# Supplementary material for: Factors Related to Smoking and Perceptions of a Behavioral Counseling and Messenger Service–Delivered Smoking Cessation Intervention for People With HIV in China: Qualitative Study
Source: JMIR Form Res. 2022 Oct 12;6(10):e35923. doi: 10.2196/35923 (PMC9607887; doi:10.2196/35923)
Supplement: Multimedia Appendix 1 [file formative_v6i10e35923_app1.doc]

**Moderator’s guide for:**

**Individual/small group interviews**

Quit For Life (QFL): Smoking Cessation Among Chinese Smokers Living with HIV

**[*Notes in these parentheses are for the moderator – not to be read out loud*]**

**Consent process**

Consent forms for interview participants must be collected in advance or before the start of the discussions.

**Introduction**

Hello. Thank you so much for coming (or for joining the discussion via WeChat/Zoom).

My name is _______ [*Moderator’s first name*] and I will lead the group discussion today. This is Dr _______ [*name to be determined*] who is working on this project and will help to take notes of our discussion.

The purpose of this study is to develop a smoking cessation program specifically for people living with HIV/AIDS who smoke [*or who used to smoke*]*.* The first step in creating the intervention is to gain insight from you and other people like you, that is other people living with HIV/AIDS who smoke [*or who used to smoke*], would like to have included in the model intervention program.

You have been asked to join us today because of your experience as a smoker/ex-smoker. For the next 60 to 90 minutes you will participating in an interview.

As the moderator, I get to ask the questions, but I am not an expert on the topic. I am simply leading the group for the researchers and help facilitate the discussion.

**As we have described in the consent form, we are not asking you to disclose any personal or sensitive information and you don’t have to speak up and say anything if you choose not to**.

Usually when we get into the conversations, plenty of ideas come up, and people can get quite involved in the discussion. So, the only rule is, give everyone their turn to speak. I will not be taking many notes. I want to listen and talk with you. Therefore, I’m going to audio-record this session. I can then go over it later and remember what all of you have discussed.

This research will be conducted in a **confidential manner** and your personal information will be guarded and not given to anyone.

This meeting is going to last approximately 60 to 90 minutes. May I begin?

**Interview guide**

**Warming up**

I’d like us to go around the room and have each of you tell us *your first name*, how long you have been smoking *(or how long have you been quit*) and how long you have been receiving HIV treatment in this clinic?

**Topic 1: Smoking behavior**

1. When did you start smoking? (How old were you at that time?)

Under what situation did you smoke for the first time? (With whom? Why?)

1. How many cigarettes do you smoke every day [on average]?

When do you usually smoke? (How do you feel when smoking?)

**Topic 2: Knowledge about harm of smoking, second hand smoke, third hand smoke**

1. What do you know about the health effects about smoking? [How does smoking influence your health? Are the effects of smoking the same for people living with HIV?]
2. What have you heard about second hand smoke or third-hand smoke? [if participants do not know what these are, then explain]

[*Second Hand Smoke*: smoke from burning tobacco products, such as cigarettes, cigars, or pipes, or that has been exhaled, or breathed out, by the person smoking.]

[*Third Hand Smoke*: the residual contamination from tobacco smoke that lingers in rooms long after smoking stops and remains on our clothes after we leave a smoky place.]

1. What are the effects of second-hand smoke on other people’s health? What are the effects of third-hand smoke on other people’s health?
2. What do you think about smoking in vehicles (i.e. private cars)?
3. How much do you know about e-cigarettes? [*Probe:* Do you use e-cigarettes? Why do you use it? Do you know anyone who use e-cigarettes? Why do they use it? If you don’t use them, have you ever thought of trying this?]

**Topic 3: Knowledge about the relationships between smoking and HIV infection and treatment for HIV**

1. What do you know about the relationship between smoking and HIV infection? [Will smoking worsen HIV situation? Will smoking negatively affect treatment outcome for HIV?]
2. (If knows about the effects)…..How did you find out about the effects of smoking and HIV? Did any healthcare workers ever talk to you about these? Other information sources?
3. In your opinion, is smoking any worse among people with HIV infection compared to people with other health conditions?

**Topic 4: Quitting smoking attempts**

1. Have you ever tried to quit smoking (or How long has it been since you quit smoking)? Would you please tell me more about that process? [*Probe:* Who persuaded you to quit smoking; When/why did you try to quit smoking?]
2. What method(s) did you use to support your quitting?
3. How many attempts did you make to quit smoking? What went wrong on the past quitting attempts? What was the reason for you to start smoking again?
4. If you are going to quit smoking in the future, what kind of help do you want to receive? (self-help materials, counselling, medication, smoking cessation hotlines)? Would you be willing to pay any money from your pocket for these services/programs/medications?
5. If you or someone you know want to quit smoking, do you know where to get help for smoking cessation counseling and services?
6. What is your impression about receiving smoking cessation services from the HIV clinic together with your HIV treatment? [*Probe:* who should deliver the counseling (doctor/nurse/other? ….. how they can be good counselor/communicator; What would make it a negative experience?]

**[BREAK for 5-10 minutes, if possible]**

**Topic 5: QFL intervention**

Now we want to discuss with you about an intervention program that we have developed to be delivered by nurse counselors. The intervention is designed to help people living with HIV quit smoking. Let me describe the intervention content and the process of intervention delivery. We will appreciate your feedback to make the intervention more useful, acceptable and relevant to the needs people living with HIV.

Brief verbal description to describe draft intervention: process, number of times to be met in person/by telephone, number of text messages, topic to be covered).

1. In your opinion, what are the best formats to deliver the QFL intervention? (e.g., in-person only, telephone only, combined in-person/telephone, text messaging/ WeChat.)
2. Let’s talk about length. What is the maximum number of contacts you would be willing to receive from a counselor? What type of contact would you prefer and for what duration?
3. Now I’d like to ask you about text or WeChat messages. How many per day would you be willing to receive?

Let me show you few messages that we are considering to use. Let me know your feedback on these.

**[Read four sample messages out loud to get subject’s reaction and run the discussion**

**Ask 1a – 1d for each of these messages]**

**Why quit? Please write down your reasons, put them at a place where you can look at every day. They will act as reminders to help you quit smoking.**

**Mood is closely related to smoking addiction. Are you happy, sad or depressed? No matter if you are a smoker or a non-smoker, life has its highs and lows, and that is what life is supposed to be. You can use "Manage my Mood" in the Home Page to manage your emotions.**

**Did you know? Quitting smoking can improve your HIV treatment success**

**Need extra help? You can call smoking cessation hotline: 12320, or go to a smoking cessation clinic for help. What are you planning to do next?**

***Questions to ask:***

**1a.** What did this message make you think of?

**1b.** Would this message be helpful in motivating you to quit smoking?

In what ways?

**1c.** Was this message easy to read?

In what ways?

**1d.** What changes would you make to this message?

1. Do you think that the QFL intervention delivery should be connected with your visit to HIV clinic to collect HIV medicine (ART) or follow up for CD4 counts? Why?
2. What other considerations should we keep in mind when developing/revising the QFL intervention? (- e.g., whether we should develop different intervention for those who want to use quit smoking medications and those who do not want to use?).
3. In addition to HIV infection, do you have any other chronic diseases, such as hypertension, diabetes or COPD?
4. Finally, how might you or your peers be convinced that participating in such an intervention would be beneficial to quit smoking as well as improving HIV situation? How might you or other PLWH know and accept this intervention? What might be the acceptable messages we should deliver to attract others to join the program? What might be the best ways to reach PLWH with these messages?

We have been calling this program QFL or Quit for Life [Chinese name] – what do you think of this name? Should we call it something else?

1. Any other issues related to QFL intervention that you think we should consider?

[*Probe:* anything else to talk to make PLWH more willing to quit, accept the program; frequency of contacts; other modes of delivery (i.e. WeChat use); *let them suggest what else to do*]

-End-
