# Supplementary material for: Factors Related to Smoking and Perceptions of a Behavioral Counseling and Messenger Service–Delivered Smoking Cessation Intervention for People With HIV in China: Qualitative Study
Source: JMIR Form Res. 2022 Oct 12;6(10):e35923. doi: 10.2196/35923 (PMC9607887; doi:10.2196/35923)
Supplement: Multimedia Appendix 2 [file formative_v6i10e35923_app2.docx]

**Multimedia Appendix 2**

**Table S1.** Frequently mentioned exploratory themes, codes, subcodes, and example quotations by smokers and former smokers with HIV in China.

| Theme and parent code | | Subcode | Additional subcode | | Quotations |
| --- | --- | --- | --- | --- | --- |
| **Exploratory theme 1.1—demographic differences: age** | | | | | |
|  | Barriers and motivators to quitting | Barriers to quit | | Helplessness | - “For me it’s not that useful. I’m already 60 years old. I think younger folks should quit smoking.” [F^a^, 60, C^b^] |
|  | Barriers and motivators to quitting | Motivators to quit | | Social influence and stigma | - “People say that I'm already so old, there’s no reason for me to smoke...And for my current condition, nothing would work out if I still smoke.” [M^c^, 58, C] |
|  | Additional concepts | E-cigarettes | | General | - “[Younger people] think it’s fun [to smoke e-cigarettes], has flavors.” [F, 44, C] |
|  | Barriers and motivators to quitting | Barriers to quit | | Risk perception | - “In my opinion, I found that currently for many people, as long as they don’t think there are any physical discomforts, they have no thoughts of quitting. Young people especially.” [F, 36, C] |
|  | Smoking behavior | Patterns of smoking habits | | When | - “When I’m chatting with friends or playing video games.” [M, 30, C] |
|  | QFL^d^ intervention | Recommendation to others | | —^e^ | - “I think the program should start with young smokers who smoke in small quantities and who can control how much they smoke every day.” [M, 46, C] |
| **Exploratory theme 1.2—demographic differences: sex** | | | | | |
|  | Barriers and motivators to quitting | Motivators to quit | | Health benefits and physical changes after quitting | - “I was smoking when I first learned about the diagnosis. Then I found out I was pregnant...so I quit.” [F, 22, C] |
|  | Sex difference | Smoking behavior | | Types of cigarettes | - “It is because ladies’ cigarettes are mostly with the mint flavor. So I want to smoke men’s cigarettes.” [F, 22, C] - “I can’t smoke cigarettes that are strong...” [F, 36, C] |
|  | Sex difference | ART^f^ use | | — | - “...men only need to take the medication once at night. For us, we have to take the medication once in the morning and once at night if we want to get married and have children...” [F, 22, C] |
|  | Sex difference | Harms of smoking | | — | - “Everyone knows that smoking is harmful, especially for women...However, men is not as harmful.” [F, 36, C] |
|  | Additional concepts | Substance use | | — | - “They rarely talked about smoking, it was all about the harm of drugs.” [F, 60, C] - “[staff in the rehabilitation center] were only in charge of helping us quit drugs. However, drug rehabilitation centers prohibit smoking.” [F, 44, C] |
|  | Sex differences | General | | — | - “Most men use smoking as a way to release their stress. They’re tired from working, and are stressed when they have a family. It is impossible for them to cry and vent in front of their wives...So smoking is the only thing left.” [M, 21, C] |
|  | Barriers and motivators to quitting | Motivators to quit | | Positive impacts on others | - “Because you have the responsibility to take care of the family, things you do are closely linked to the future of the family.” [M, 24, Q^g^] |
| **Exploratory theme 1.3—demographic difference: education level** | | | | | |
|  | Knowledge | Second- and third-hand smoke | | — | - “I don’t know much about them.” [F, 22, Q, elementary school] - “I know second-hand smoke but not third-hand smoke.” [M, 24, C, senior high school] - “It means inhaling the smoke that someone else in your home is exhaling. Or you are inhaling the smoke that others exhaled in public spaces. Second-hand and third-hand smoke are more harmful than smoking one’s own cigarette.” [F, 60, C, senior high school] |
|  | Barriers and motivators to quitting | Barriers to quit | | Risk perception | - “It’s definitely bad for my lungs...After all, I'm addicted to smoking, at least at the moment smoking does not cause any harm for me so far, so I did not care too much.” [F, 44, C, junior high school] - “Yes it does, people say it’s carcinogenic and affects my lungs. However, since I’m smoking, I shouldn’t think of those things too much.” [M, 21, C, technical secondary school] |
|  | Knowledge | Health effects | | — | - “I think smoking would [effect my health], and that’s why I try to control myself to smoke less.” [M, 24, C, undergraduate] |

^a^F represents participants who report to be female.

^b^C represents participants who report to be current smokers.

^c^M represents participants who report to be male.

^d^QFL: *Quit for Life*.

^e^No additional subcodes.

^f^ART: antiretroviral therapy.

^g^Participants who report to be former smokers and have quit smoking.
